# Trials and Tribulations with Applications for Dental Postgraduate Courses

**Published:** 1988-11

**Authors:** B. G. H. Levers, C. Scully, R. W. Matthews, J. P. Shepherd

**Affiliations:** University Department of Oral Medicine, Surgery and Pathology, Bristol Dental School, Lower Maudlin Street, Bristol BS1 2LY; University Department of Oral Medicine, Surgery and Pathology, Bristol Dental School, Lower Maudlin Street, Bristol BS1 2LY; University Department of Oral Medicine, Surgery and Pathology, Bristol Dental School, Lower Maudlin Street, Bristol BS1 2LY; University Department of Oral Medicine, Surgery and Pathology, Bristol Dental School, Lower Maudlin Street, Bristol BS1 2LY


### Trials and Tribulations with Applications from non-UK Nationals for Dental Postgraduate Taught Courses in UK

**Dr B. G. H. Levers MSc PhD**
**Professor C. Scully BSc BDS MBBS PhD FDSRCPS MRCPath**
**Dr R. W. Matthews MDS PhD**
**Mr. J. P. Shepherd MSc BDS FDSRCS**
University Department of Oral Medicine, Surgery and Pathology, Bristol Dental School, Lower Maudlin Street, Bristol BS1 2LY

There is an increasing demand for postgraduate dental education in the UK from non-UK nationals. It appears to be a common experience that the administration of such applications is fairly complicated. We have analysed our experiences in relation to overseas applications to enter taught courses for the MSc in Medical Sciences in the University Department of Oral Medicine, Surgery and Pathology at Bristol.

All students are taught a common core of dentally-related subjects and also follow one of six specialised topics. They undertake a short research project and submit a dissertation based on this work. The MSc is awarded on the results of examination of the dissertation, clinical work, essay and multiple choice papers.

The courses were first advertised in January 1984 and we have analysed the applications for four intakes.

This department has received 199 written enquiries from non-UK nationals.

Oral Surgery was the most popular choice of specialty (46.2% of enquiries), followed by periodontology (19.6%).

Enquiries were received from applicants from 34 different countries, the majority originating from the Middle East (49.6%) and the Indian sub-continent (11.7%).

From the total of 199 enquiries, 117 (58.8%) proved to be initial enquiries only and were not followed up by firm applications.

Fifty-one applications (25.6%) were acceptable, although 14 of these (5.5%) withdrew later, sometimes because of a failure to obtain the necessary funding, but usually with no defined reason given. A further 11 acceptable applicants (5.5%) failed to reply to further communications, even after provisional acceptance.

Of the remaining 31 applications, 24 (12.1%) were rejected.

Rejection decisions were made on the basis of the applicant's academic merit and the overall quality of the applications plus referees' reports, or because of their low standard of written English.

The fate of the remaining 7 applicants (3.5%) has yet to be decided.

The largest single group of firm applicants were from Iraq and Iran. This may well reflect the current political situation in that area, as several of the applicants from that area were already doing courses in the UK and wished to remain. The majority of other applications were from Third World or somewhat underdeveloped areas and, notably, very few were received from Western Europe, North America, Scandinavia or Eastern bloc countries.

The most notable feature is that 58% of enquiries were not followed up by the applicant despite a prompt response in which we posted a package containing details of the courses, an application form and information about facilities in the Dental School, University and the City.

In summary, only 41.2% of all applications (82 of 199), could be regarded in any way as serious, and it was impossible to identify these at an early stage. Only approximately 24% of firm applications were deemed acceptable.

The majority of applications appeared, on paper, to be reasonable, as did the referees' reports. Difficulties arose when applicants failed to respond to our written communications or later denied knowledge of our response. The low rate of further response from initial applicants suggests they may be making multiple enquiries in the UK and elsewhere. This creates difficulties in administration, since the University feels morally obliged to honour acceptance of candidates who may not themselves honour the agreement.

A clinical discipline is strictly limited in the number of postgraduates that can be taken on, since space is limited and undergraduate and junior hospital staff teaching must not be impeded. We need, therefore, a method of determining the seriousness of applications at a much earlier stage, and it may be that UK Universities should look to the establishment of a co-ordinating centre for non-UK applications for postgraduate dental studies, or at least to some form of inter-Varsity communication.

### REFERENCES

LEVERS, H., SCULLY, C., MATTHEWS, R. and SHEPHERD, J. (1987) Trials and tribulations with applications from non-UK nationals for dental postgraduate taught courses. Letters to Editor, *Br Dental J* **162**, 138.

